# Diagnostic, Prognostic, and Therapeutic Value of Circulating miRNAs in Heart Failure Patients Associated with Oxidative Stress

**DOI:** 10.1155/2016/5893064

**Published:** 2016-06-09

**Authors:** Md Sayed Ali Sheikh, Umme Salma, Baohai Zhang, Jimei Chen, Jian Zhuang, Zhu Ping

**Affiliations:** ^1^Department of Cardiovascular Surgery, Guangdong Cardiovascular Institute, Guangdong General Hospital, Guangdong Academy of Medical Sciences, Guangzhou 510100, China; ^2^Department of Cardiology, Hunan Traditional Chinese Medical University, International Education Institute, Changsha, Hunan 410208, China; ^3^Department of Gynecology and Obstetrics, Xiangya 3rd Hospital, Central South University, Changsha, Hunan 410013, China; ^4^Department of Cardiology, Affiliated Hospital of Jiangsu University, Zhenjiang 212001, China

## Abstract

Heart failure is a major public health problem especially in the aging population (≥65 years old), affecting nearly 5 million Americans and 15 million European people. Effective management of heart failure (HF) depends on a correct and rapid diagnosis. Presently, BNP (brain natriuretic peptide) or N-terminal pro-brain natriuretic peptide (NT-proBNP) assay is generally accepted by the international community for diagnostic evaluation and risk stratification of patients with HF. However, regardless of its widespread clinical use, BNP is still encumbered by reduced specificity. As a result, diagnosis of heart failure remains challenging. Although significant improvement happened in the clinical management of HF over the last 2 decades, traditional treatments are ultimately ineffective in many patients who progress to advanced HF. Therefore, a novel diagnostic, prognostic biomarker and new therapeutic approach are required for clinical management of HF patients. Circulating miRNAs seem to be the right choice for novel noninvasive biomarkers as well as new treatment strategies for HF. In this review, we briefly discuss the diagnostic, prognostic, and therapeutic role of circulating miRNAs in heart failure patients. We also mentioned our own technique of extraction of RNA and detection of circulating miRNAs from human plasma and oxidative stress associated miRNAs with HF.

## 1. Introduction

Heart failure (HF) is defined as the inability of the heart to pump sufficient blood to meet the body demands. Heart failure is a major public health problem especially in the aging population (≥65 years old), affecting nearly 5 million Americans and 15 million European people. HF is associated with high morbidity and reduced life expectancy, with 5-year mortality of newly diagnosed HF as high as 50% and 10-year survival of 26.7% [1, 2]. Systolic and diastolic dysfunctions are considered the most important underlying pathology of HF and it mainly occurred due to ischemic heart disease (coronary artery disease, myocardial infarction), uncontrolled hypertension, idiopathic dilated cardiomyopathy (IDCM), hypertrophic cardiomyopathy (HCM), chronic myocarditis, and valvular heart diseases [3, 4].

Effective management of HF depends on a correct and rapid diagnosis. Presently, BNP (brain natriuretic peptide) or N-terminal pro-brain natriuretic peptide (NT-proBNP) assay is generally accepted by the international community for diagnostic evaluation and risk stratification of patients with HF. However, regardless of its widespread clinical use, BNP is still encumbered by reduced specificity. As a result, diagnosis of HF remains challenging. Although significant improvement happened in the clinical management of HF over the last 2 decades, traditional treatments are ultimately ineffective in many patients who progress to advanced HF. Therefore, a novel diagnostic, prognostic biomarker and new therapeutic approach are required for clinical management of HF patients. Circulating miRNAs seem to be the right choice for novel noninvasive biomarkers as well as new treatment strategies for HF [1, 5].

Microribonucleic acids (miRNAs) are highly specific, endogenous, small (~22 nucleotides), single-stranded, noncoding RNAs that regulate the rate of protein synthesis at the posttranscriptional level by binding to the 3′-untranslated region (3′-UTR) through altering the stability of the targeted mRNAs. Recently, it has been well established that miRNAs are critically regulating all the biological functions such as development, metabolism, cellular differentiation, proliferation, migration, secretion, excitation, conduction, aging, apoptosis, stem cell regulation, and immune function of the cell types relevant to the cardiovascular system such as endothelial cells and cardiac myocytes and conductive cells and smooth muscle and inflammatory cells and fibroblasts. Moreover, miRNAs are directly involved in many cardiovascular disease conditions [6, 7].

However, presence of miRNAs in body fluid is called circulating miRNAs. Circulating miRNAs have some useful characteristics as biomarkers, since they are highly constant and easily detectable in the peripheral circulation. Several research groups established that altered circulating miRNAs levels have been associated with different forms of heart disease, including ventricular septal defect (miR-155-5p, miR-222-3p, and miR-498) [8], atrial and ventricular arrhythmia (miR-1, miR-26, and miR-328) [9–13], hypertension (miR-296, miR-133b, and miR-625) [14], coronary artery disease (miR-133a, miR-208a, and miR-126) [15, 16], as well as, in our previous study, miR-149, miR-424, and miR-765 [17, 18], acute coronary syndrome (miR-1, miR-133a, and miR-208a) [19], acute myocardial infarction (miR-208b, miR-1, and miR-133) [20–23], and heart failure (miR-423-5p, miR-320a, and miR-22) [24, 25].

It has been well known that oxidative stress induced cardiac cell injury through expression changes of multiple genes plays a critical role in the pathogenesis of various types of heart diseases and there is a strong correlation between oxidative stress markers and HF. Several factors are associated with oxidative stress; among them increased reactive oxygen species (ROS) levels or decreased antioxidant defenses are responsible for disease progression [26, 27]. ROS is a collective term which includes hydrogen peroxide (H_2_O_2_), superoxide anion O_2_^−•^, and hydroxyl radicals (^•^OH). ROS can be produced within living cells by several potential sources such as mitochondria, plasma membrane bound NADPH oxidases (NOXs), endoplasmic reticulum (ER), and different enzymes involved in redox reactions such as xanthine oxidases (XOs), lipoxygenases, peroxidase, cytochromes, mono- and dioxygenases, and uncoupled nitric oxide synthases (NOSs) [28–31].

In experimental study, it has been established that mitochondrial DNA deletions contribute to the phenotype of systolic heart failure through increased mitochondrial ROS [32]. Congestive heart failure is related to intrinsic alterations of mitochondrial oxidative phosphorylation which lead to elevated myocardial cytosolic free ADP. ATP sensitive K^+^ (KATP) channels act as metabolic sensors that are essential for maintaining coronary blood flow and for mediating the response of the myocardium to oxidative stress. As a result, in the failing heart the balance between myocardial ATP demands and oxygen supply is significantly dependent on functioning KATP channels. However, KATP channels' function is critically regulated by miR-9a-3p [33, 34].

A recent study demonstrated that miRNAs exist in the heart mitochondria which significantly regulates mitochondrial gene expression and prevents ROS induced cardiomyocyte injury [35]. Nicotinamide adenine dinucleotide phosphate oxidase (NOX) proteins produce ROS involved in redox signaling pathway and directly linked with development of HF. Accumulating evidence supports mainly beneficial effects of Nox4 in the cardiovascular system. Overexpression of Nox4 reduced angiotensin II-induced high blood pressure, protects endothelial dysfunction and atherosclerosis, also regulating oxidative stress and apoptosis in cardiac myocytes, and prevents the development of HF [36–39]. However, miR-188, miR-146a, and miR-25 significantly regulated the expression of NOX4 and protected oxidative/nitrative stress induced endothelial and myocardial dysfunction [40–42].

In this review, we mainly focus on more recent clinical data regarding circulating miRNAs and their potential value in diagnosis, prognosis, and therapeutic targets for heart failure patients. In addition, we also discuss our own technique of extraction of RNA and detection of circulating miRNAs from human plasma and oxidative stress associated miRNAs with HF.

## 2. Discovery of MicroRNA to Circulating MicroRNA

**Figure 1:**
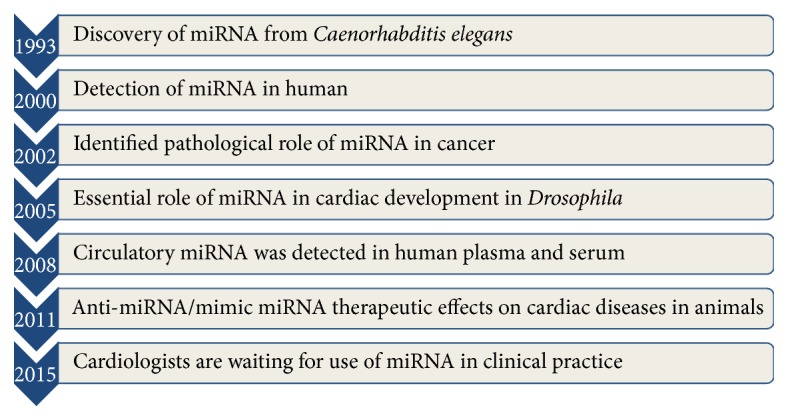


MicroRNA was first discovered in 1993 by Lee et al. [43] and Wightman et al. [44] from the nematode* Caenorhabditis elegans* and 7 years later it was identified in humans. In 2002, Calin et al. firstly recognized the pathological role of miR-15 and miR-16 in the development of chronic B-cell leukemia [45] (Figure 1). In 2005, Kwon et al. firstly established the role of miR-1 in cardiac development in* Drosophila* and it is critically regulated by the Notch signaling pathway (Notch 1 receptor) [46]. Just one year later, Van Rooij et al. through northern blot analysis from cardiac tissue demonstrated that several stress-responsive miRNAs were significantly dysregulated in cardiac hypertrophy and HF in both mice and humans. Moreover, they also reported that in particular miR-195 was remarkably upregulated in idiopathic end-stage failing human hearts and plays an important role in cardiac remodeling in transgenic mice [47].

According to Mitchell et al., in 2008, the presence of miRNA was detected in plasma and serum of prostate cancer patients and healthy subjects. They also reported that circulating miRNAs are remarkably stable to incubation at room temperature for up to 24 h or to subjecting them to up to eight cycles of freeze-thawing and are highly protected from endogenous RNase activity.

Very importantly, when synthetic miRNAs* (cel-miR-39, cel-miR-54, and cel-miR-238)* were added to plasma prior to RNase denaturing solution, they were rapidly degraded (<2 min)—suggesting that exogenous miRNAs are not stable to RNase activity, but endogenous plasma miRNAs are resistant to RNase activity because they are normally attached to microvesicles or exosomes and also form protein-miRNA complexes in extracellular fluids [48]. Several other studies also established that circulating miRNAs remain stable after being subjected to severe conditions that would usually degrade most RNAs, such as boiling, very low, or high pH levels. Besides, recent studies have evaluated that miRNAs are preserved in archived 10-year-old human serum samples [49, 50].

More recently, miRNAs have been easily detected in a wide range of bodily fluids, including urine, saliva, tears, seminal fluid, cerebrospinal fluid, pleural fluid, peritoneal fluid, amniotic fluid, and breast milk. Therefore, the discovery of miRNAs in body fluids opens up the possibility of using them as noninvasive diagnostic and prognostic biomarkers in cardiovascular diseases including HF [51, 52]. Though last two decades huge research happened in microRNAs, the origins of circulating miRNAs are largely unknown. Some studies have proposed that they are secreted in membrane-bounded-vesicles (apoptotic bodies, microvesicles, and exosomes), while others mentioned that they are secreted in vesicle-free medium but associated with protein-miRNA complexes (AGO2, NPM1, and HDL). It has also been hypothesized that circulating miRNAs are released from various healthy or disease tissues such as heart, lung, liver, kidney, and brain. According to miRBase data line (http://www.mirbase.org/), up to date 2588 mature miRNAs were exploded in human; among them more than 200 miRNAs were identified as a circulating miRNAs [4, 5].

## 3. Biogenesis of Circulating miRNAs

In the nucleus, microRNA genes are transcribed by RNA polymerase II into many hundreds or thousands of nucleotides in length called primary miRNAs (pri-miRNAs), which are folded into characteristic hairpin structures. Then, pri-miRNAs are cleaved by Drosha, RNase III enzyme, in association with DGCR8, RNA-binding protein, to form smaller, 70- to 100-nucleotide-long, preliminary miRNAs (pre-miRNAs). Subsequently, pre-miRNAs are actively transported to the cytoplasm by the Ran-GTP dependent transporter in association with exportin-5 [7, 53].

**Figure 2:**
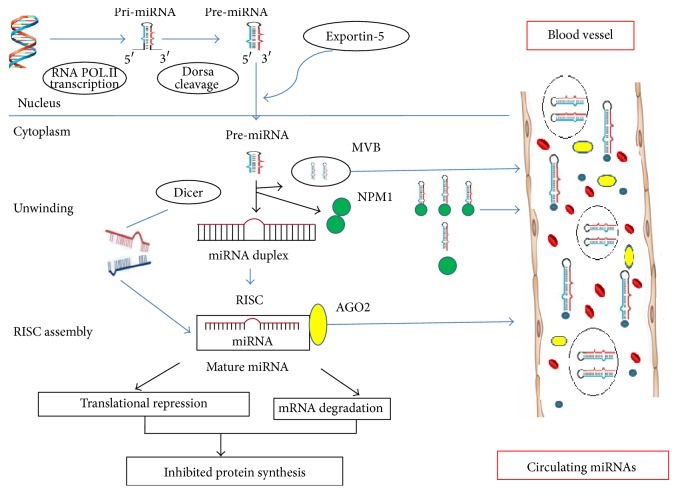
Biology and mechanism of circulatory miRNAs.

In the cytoplasm, the pre-miRNAs are cleaved by Dicer (another RNase III enzyme) and processed into double-stranded, immature miRNA (miRNA:miRNA^*∗*^) duplexes which are approximately 22 nucleotides in length. Afterward, the duplex is unwound and assembled into the RNA-induced silencing complex (RISC) by associating with argonaute proteins; the mature miRNA negatively regulates gene expression through translational repression or mRNA degradation, according to the sequence complementarity between the miRNA and the target mRNA. Moreover, miRNA and mRNA interaction is thought to be an ideal Watson-Crick base-pairing of nucleotides 2 to 8 (seed region) at the 5′-end of the miRNA, whereas nucleation sites usually present on the 3′-untranslated region (3′-UTR) of the targeted mRNA. Pre-miRNAs can be attached to MVBs or other RNA-binding proteins (NPM1, AGO2, or HDL); after fusion with the plasma membrane, circulating miRNAs enter the bloodstream. MiRNAs can also be released by cells through exosomes, apoptotic bodies, and other vesicular-like bodies [7, 51, 53]. More details are in Figure 2.

## 4. Extraction of RNA and Detection of Circulating miRNAs from Human Plasma

In our previous experiments [17, 18, 62], we enrolled 300 human subjects with the acute myocardial infarction (AMI), coronary artery disease (CAD), and AMI with HF and healthy controls and we used the following techniques and achieved the best result.

### 4.1. RNA Isolation

**Figure 3:**
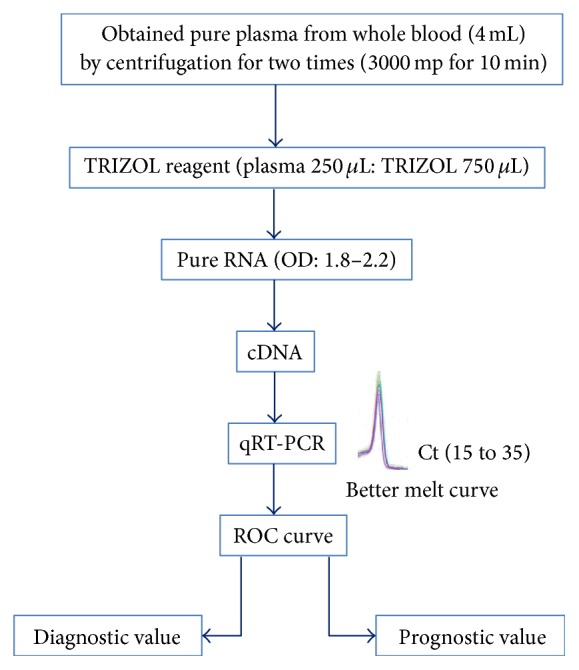


RNA isolation is as follows:

1. Total RNA was isolated from human plasma and mice plasma by using a phenol-based TRIZOL reagent. In short, 250 *μ*L of plasma was mixed briefly with 750 *μ*L of TRIZOl, incubated for 5 min at room temperature (RT), and then mixed with 200 *μ*L chloroform, incubated for 3 min at RT.
2. The aqueous phase, interphase, and organic phase were separated by centrifugation at 12,000 rmp for 15 min at 4°C.
3. The upper aqueous phase was collected and subsequently mixed with 500 *μ*L of 100% isopropanol and incubated at −20°C for 16 h and after that centrifuged at 13000 rmp for 15 min at 4°C for precipitation.
4. RNA samples were washed 3 times with 500 *μ*L of 75% ethanol and centrifuged again at 7500 rmp for 10 min at 4°C.
5. Finally, supernatants were eliminated and dried for 5 min. Then, RNA samples were dissolved in 30 *μ*L of RNase-free (DEPC) water and incubated overnight at 4°C.
6. Afterward, the concentration and purity of RNA were determined spectrophotometrically and stored at −80°C for future use (Figure 3).

### 4.2. Detection of Circulating miRNAs

Real-time quantitative reverse-transcription PCR analysis was carried out to examine the expression of specific miRNAs in plasma. A total 4 *μ*L of pure RNA (OD, 1.8–2.2; nucleic acid concentration, 50–500 *μ*g) was reverse-transcribed to cDNA at 42°C for 30 minutes. Subsequently, 2 *μ*L of cDNA was used as the template in real-time quantitative PCR reaction. MiR-156a and U6 were used as the normalization control. The Ct values from qRT-PCR assays between 15 and 35 were considered to be expressed.

To decrease the possible errors resulting from qRT-PCR assays, in our previous study we used three potential endogenous control miRNAs (U6, cel-miR-39, and miR-156a); among them we had chosen miR-156a as a standard inner control. Depending on our own experience, the use of synthetic mimic miR-156 instead of commonly used cel-miR-39 during RNA extraction from plasma of AMI or CAD patients obtained better quality of total RNA (OD ratio: 1.8–2.2, nucleic acid concentration: 50–500 *μ*g). Furthermore, with the use of endogenous miR-156 instead of commonly used U6 for endogenous control during real-time PCR, we achieved more reliable results (Ct value: 18–25) [17, 18, 62].

## 5. Circulating miRNAs as a Potential Biomarker for HF Patients

Recently, several research groups suggested that circulating miRNAs might be useful as stable blood-based biomarkers in HF patients. Tijsen et al. found that 6 circulating miRNAs (miR-423-5p, miR-18b, miR-129-5p, miR-1254, miR-675, and miR-622) were upregulated in patients with HF, among which miR-423-5p was most significantly related to the clinical diagnosis of HF. Moreover, the predictive power of miR-423-5p was also high within the dyspnea population, upon comparing HF and non-HF cases. Furthermore, circulating miR-423-5p was significantly upregulated in systolic HF patients and rat HF model and closely associated with N-terminal pro-brain natriuretic peptide (NT-proBNP) and ejection fraction (EF) [24, 54].

Cardiac myocyte-associated plasma miR-499 was highly elevated in patients with acute heart failure (AHF) as compared with control subjects, whereas no significant changes were observed in diastolic dysfunction and it was not affected by a wide range of clinical parameters, including age, sex, body mass index, kidney function, systolic blood pressure, and white blood cell count. Furthermore, plasma level of the liver-specific miR-122 was not only increased in hepatic injury but also significantly increased in AHF patients, possibly reflecting hepatic venous congestion [20]. Goren et al. demonstrated that serum levels of miR-320a, miR-22, and miR-92b were significantly elevated in systolic HF patients and correlated with important clinical parameters such as elevated serum NT-proBNP levels, a wide QRS, and dilatation of the left ventricle and left atrium [24].

The plasma miRNA expression levels of chronic HF patients were compared to healthy subjects using a microRNA array, and it was noticed that plasma miR-361-5p levels were significantly decreased in all individuals with chronic HF. This result suggested plasma miR-361-5p as a potential, novel biomarker for chronic HF patients [63].

A clinical study examined the miRNA expressions in peripheral blood mononuclear cells (PBMCs) from chronic HF patients and found that miR-107, miR-139, and miR-142-5p were markedly downregulated in both non-ischemic dilated cardiomyopathy (NIDCM) and ischemic cardiomyopathy (ICM) patients compared to healthy subjects. Furthermore, miR-142-3p and miR-29b levels were highly upregulated in NIDCM while miR-125b and miR-497 levels were downregulated in ICM patients. Therefore, miRNAs in peripheral blood mononuclear cells may be used as biomarkers for diagnosis of chronic HF patients [64].

Circulating miR-155 levels were significantly decreased in patients with chronic HF and strongly associated with ventricular arrhythmia and angiotensin receptor type 1 single nucleotide polymorphism 1166A/C [65]. It has been demonstrated that muscle-specific miR-133 expression is associated with signs of HF in patients undergoing coronary artery bypass surgery. According to New York Heart Association (NYHA) functional classification of HF, miR-133 expression decreased significantly with increased severity of HF. Additionally, patients with NT-proBNP levels >1,800 pg/mL showed a 25% decrease in miR-133 expression compared to patients with levels <300 pg/mL, indicating its potential as a biomarker for HF [55].

A comprehensive microRNA (miRNA) and messenger RNA (mRNA) analysis was performed on plasma and myocardial specimens from HF patients, chronic obstructive pulmonary disease (COPD) patients, and healthy controls and found that miR-103, miR-142-3p, miR-30b, and miR-342-3p levels were differentially expressed between HF and controls and COPD and other breathless patients. Though individually NT-proBNP was most significant in predicting HF and exhibited greater sensitivity and specificity, combining microRNA levels with NT-proBNP may add diagnostic value. Furthermore, the expressions of circulating microRNAs (miR-30b, miR-103, miR-199a-3p, miR-23a, miR-27b, miR-324-5p, miR-342-3p, and miR-142-3p) were significantly altered in end-stage HF patients. Moreover, although the miRNAs are more sensitive than mRNAs for diagnosis of heart failure patients, combined miRNA and mRNA profiling may have better value for diagnosis and prognosis of end-stage cardiomyopathy patients [56, 66].

The plasma level of miR-126 was remarkably decreased in congestive heart failure (CHF) patients and negatively correlated with age, logBNP, and NYHA class and could be a useful biomarker for CHF [57]. The serum expression levels of miR-210 and miR-30a were significantly elevated in CHF patients compared to healthy subjects and correlated with NT-pro-BNP. In addition, these miRNA levels were higher in patients with EF >40% than in those with EF <40%, which might serve as a potential biomarker for HF [58].

Recent study identified that three cardiac fibroblast-derived miRNAs (miR-660-3p, miR-665, and miR-1285-3p) were found obviously elevated in heart and plasma during CHF and significantly correlated with left ventricular ejection fraction (LVEF), holding promise as potential diagnostic biomarker for CHF. These three circulating miRNAs may be used as potential biomarkers for diagnosis of CHF patients [67].

Serum level of miR-1 was markedly downregulated in patients with symptomatic HF and its expression reduced with severity of NYHA class, as well as being negatively correlated with NT-proBNP concentration in patients in NYHA class II/III. Upregulation of miR-21 was found in all patients, independent of HF severity, and significantly correlated with galectin-3 concentration. As a result, alteration of miR-1 and miR-21 expression may be essential for the development of HF; miR-1 might become a biomarker for diagnosis of HF [68]. The expression of baseline plasma miR-30d level is significantly related to response to cardiac resynchronization therapy (CRT) in HF patients with dyssynchrony (HFDYS). Upregulation of miR-30d in cultured cardiomyocytes is highly correlated with areas of increased wall stress in HFDYS and led to cardiomyocyte growth and protected against apoptosis by targeting the mitogen-associated kinase 4. In addition, miR-30d plasma level is negatively correlated with high-sensitivity troponin T in HFDYS patients. Therefore, plasma miR-30 is an essential biomarker for HF patients [69].

Very interestingly, myocardial biopsy specimens collected from Chinese patients presenting with recent HF were compared with a group of patients without HF undergoing routine cardiac surgery and it was found that miR-1, miR-21, miR-23, miR-29, miR-130, miR-195, and miR-199 expressions were evidently increased in the HF group as compared to those without HF. Furthermore, related mRNAs (casp3, coll I, coll III, and TGF) were also significantly upregulated in the HF group. These miRNAs may be used as an early diagnostic biomarker for CHF patients and possibly future therapeutic targets [70].

One study reported that the expressions of serum miR-21, miR-378, and miR-940 levels were considerably upregulated in response to an acute exhaustive exercise in CHF patients while the rest were not changed. However, no robust correlation was recognized between changes of these miRNAs and exercise capacity, muscle damage, or inflammation. Therefore, circulating miRNAs may be used as a diagnostic or prognostic biomarker of exercise adaptation in CHF patients [71]. More recently, it has been demonstrated that altered circulating miR-199a-3p levels are the best predictor of early worsening renal function (WRF) in AHF patients [72].

The expression of miR-214 was significantly increased in the serum of CHF patients, as well as in hypertrophic and failing hearts of humans and mice. On the contrary, upregulation of miR-214 markedly reduced angiogenesis of human umbilical vein endothelial cells (HUVECs) by targeting XBP1. Therefore, miR-214 plays an important role in the control/inhibition of cardiac angiogenesis which is associated with HF [73]. Plasma miRNAs (miR-16, miR-20b, miR-93, miR-106b, and miR-223) levels were most robustly changed in rats with hypertension-induced HF and also highly correlated with circulating BNP. These plasma microRNAs could potentially serve as biomarkers of therapeutic efficacy and disease progression in hypertension-induced HF [74].

Nair et al. demonstrated miRNA screening in isolated diastolic dysfunction with preserved systolic function to discover promising candidate miRNAs. They found that circulating miRNAs (miR-454, miR-500, miR-1246, and miR-142-3p) expressions levels were significantly altered in diastolic dysfunction patients. In addition, circulating miR-454 and miR-500 were decreased, while miR-1246 was increased in diastolic dysfunction. Furthermore, circulating miR-142-3p level was significantly reduced while miR-124-5p level was markedly elevated in stable compensated dilated cardiomyopathy patients. Therefore, these circulating miRNAs may serve as new diagnostic biomarkers and be suggested as therapeutic targets for drug discovery of HF patients [75].

Very recently, a translational perspective study was carried out on three patient cohorts, no heart failure (no-HF), HF with reduced ejection fraction (HFrEF), and HF with preserved ejection fraction (HFpEF), using Taqman miRNA arrays. The serum levels of miR-30c, miR-146a, miR-221, miR-328, and miR-375 were differentially expressed in HFpEF and HFrEF, and their expression levels were also different between HF and no-HF. It was summarized that combinations of two or more miRNAs with BNP are essential biomarkers for diagnosis of HF and also helpful in the differentiation of HFpEF from HFrEF compared with using BNP alone [1]. In a study group, pericardial fluid (PF) was collected from HF patients during open-heart surgery and more than 2 hundred microRNAs were detected in PF. But 5 miRNAs (miR-21-5p, miR-451a, miR-125b-5p, let-7b-5p, and miR-16-5p) were highly expressed in PF. However, the overall miRNA expressions of the cardiac patients were nearly similar despite the differences in disease aetiologies and HF stages. They concluded that miRNAs may act as endocrine and paracrine signaling factors by mediating the local crosstalk between cardiac cells [76].

The incidence of ischemic HF in post-AMI patients is increasing. The serum level of miR-192 was markedly elevated in AMI patients with development of ischemic HF. Moreover, miR-194 and miR-34a expression levels were not only upregulated but also significantly correlated with left ventricular end-diastolic dimension 1 year after AMI. Therefore, circulating p53-responsive miRNAs (miR-192, miR-194, and miR-34a) are useful predictive indicators of heart failure after AMI [59]. A prospective, nonrandomized study was performed in 81 patients with symptomatic (NYHA functional class III or IV) HF eligible for CRT. At 12-month follow-up, 55 patients (68%) were considered responders and 26 were considered nonresponders to CRT (32%). Among responders, five circulating miRNAs (miR-26b-5p, miR-145-5p, miR-92a-3p, miR-30e-5p, and miR-29a-3p) were significantly increased as compared with nonresponders, and these miRNAs inversely correlated with NT-proBNP and directly correlated with EF. Besides, in responders, reverse remodeling is associated with favourable changes in miRNAs that regulate cardiac fibrosis, apoptosis, and hypertrophy. This study indicated that serum miRNAs may be used as a potential prognostic biomarker and a new therapeutic target for HF [60].

In a large cohort, clinical study investigated circulating miRNAs expression patterns in patients with stable and advanced HF before and at different time points after a left ventricular assist device (LVAD) implantation. Circulating miR-1908 was 2.0- and 2.1-fold and miR-1180 was 4.5- and 4.0-fold upregulated in patients with advanced HF and in patients with stable HF compared with healthy controls (NFs), respectively. Moreover, the cardiac-specific circulating miR-208b, miR-208a, and miR-499 and the muscle-specific miR-1-1 and miR-133b were 143-fold, 78-fold, 28-fold, 18-fold, and 21-fold higher in advanced HF at LVAD implantation compared with NFs. Surprisingly, the miRNA changes in advanced HF reversed 3 and 6 months after LVAD support. It was suggested that circulating miRNAs serve as excellent diagnostic and prognostic biomarkers and also have therapeutic potential for HF patients [3].

Morley-Smith et al. retrospectively obtained 53 serial plasma and 20 ventricular myocardial samples from 19 patients with severe advanced heart failure who underwent HeartMate II LVAD implantation. By using microarray assay, they found that expressions of circulating miR-483-3p levels were markedly sustained upregulated with LVAD support, with median fold changes from baseline of 2.17, 2.27, 1.87, and 2.82 at 3, 6, 9, and 12 months, respectively, while baseline plasma miR-1202 identified good versus poor LVAD responders. In addition, NT-proBNP levels were inversely correlated with duration of LVAD support. As they suggest, these two circulating miRNAs are novel prognostic biomarkers for HF patients [61] (Table 1).

## 6. Therapeutic Role of miRNAs in HF

Acute and chronic stress to the heart results in a pathological remodeling response accompanied by cardiomyocyte hypertrophy, fibrosis, pump failure, and myocyte degeneration and apoptosis, which leads to HF and sudden cardiac death. Therapeutic inhibition of cardiac-specific miR-208a via subcutaneous delivery of anti-miR-208a during hypertension-induced HF in Dahl hypertensive rats dose-dependently prevents pathological cardiac remodeling and improves cardiac function with survival rate [77]. Intravenous delivery of locked nucleic acid- (LNA-) modified anti-miR-15 dose-dependently reduces infarct size, cardiac fibrosis, and pathological cardiac remodeling and enhances cardiac function in both mice and pigs [78].

The level of miR-1 was downregulated in a chronic rat HF model and its expression was restored to normal levels during reverse remodeling by sarcoplasmic reticulum calcium ATPase 2a (SERCA2a) gene therapy through an Akt/FoxO3A-dependent pathway, which was also associated with normalized sodium-calcium exchanger 1 (NCX1, functional target of miR-1) expression and significantly improved cardiac function [79]. Left ventricular assist devices (LVADs) are being used in patients with HF. Plasma *α*-1-antichymotrypsin (ACT) levels were upregulated in HF patients as compared with healthy subjects and normalized by 6 months of LVAD support. MiR-137 directly targeted ACT, thereby indicating that ACT and miR-137 play an important role in the pathophysiology of HF and reverse remodeling during mechanical support in chronic HF [80]. Trimetazidine (TMZ) improves right ventricular (RV) function and decreases apoptosis and fibrosis in RV myocardial cells (RVMCs) by increasing miR-21 expression in vitro and in vivo [81]. Circulating levels of miR-16, miR-20b, miR-93, miR-106b, miR-223, and miR-423-5p were significantly upregulated in response to hypertension-induced HF, whereas this effect was blunted in response to treatment with anti-miR-208a as well as an ACE inhibitor and prevented progression of hypertension-induced heart HF [58].

The miR-24 is enriched in cardiac endothelial cells and considerably increased after cardiac ischemia. Besides, miR-24 significantly induced endothelial cell apoptosis and markedly impaired angiogenesis. Inhibition of endothelial miR-24 significantly reduced myocardial infarct size via prevention of endothelial apoptosis and enhancement of vascularity after AMI through targeting of the endothelium-enriched transcription factor GATA2 and the p21-activated kinase 4 (PAK4), which led to preserved cardiac function and was closely related to HF [82].

In a rat aortic stenosis model and end-stage HF patients, the expressions of miR-24 were significantly upregulated in failing cardiomyocytes, which is a suppressor of JP2 expression. Junctophilin-2 (JP2) connects the sarcoplasmic reticulum (SR) to the cell membrane, including T-tubules (TT), forming structural units for excitation-contraction (E-C) coupling in cardiomyocytes. Bioinformatic analysis predicted two prospective binding sites of miR-24 in the 3′-untranslated regions of JP2 mRNA. Therefore, miR-24 and JP2 have a strong relationship between the upstream hypertrophy/HF signals and defective E-C coupling, and suggests a new therapeutic option for the treatment of HF [83, 84].

Furthermore, yes-associated protein (YAP) promotes cardiomyocyte growth in postnatal hearts. The miR-206 regulates YAP-induced cardiac hypertrophy and survival during ischemia/reperfusion injury by silencing Forkhead box protein P1, which is related to HF [85]. Recently, it has been demonstrated that circulating miR-340 was significantly upregulated in failing human hearts because of dilated cardiomyopathy. On the contrary, knockdown of miR-340 using antagomir remarkably attenuated cardiac eccentric hypertrophy and HF via target gene dystrophin (DMD) [86].

The expressions of miR-212 and miR-132 were significantly upregulated in cardiomyocyte by hypertrophic stimuli through hyperactivation of prohypertrophic calcineurin/NFAT signaling pathway and an impaired autophagic response. However, pharmacological inhibition of miR-132 by intravenous antagomir injection rescues cardiac hypertrophy and pressure-overload-induced HF in mice, offering a novel therapeutic approach for cardiac failure [87].

Six members of the miR-17-92 cluster are strongly associated with aging, among which miR-18a, miR-19a, and miR-19b were significantly downregulated in aged cardiomyocytes and hearts of old failure-prone mice and also directly linked with elderly HF patients. In addition, miR-18 and miR-19 regulated their targets' connective tissue growth factor (CTGF) and thrombospondin-1 (TSP-1) expression, respectively, and prevent age-related remodeling in the heart. It was suggested that these miRNAs may be used as potential new therapeutic targets for the modulation of aging-induced cardiac remodeling in geriatric patients [88].

MicroRNAs profiling and differential expressions analysis were performed in transplanted hearts of HF patients with LVAD implantation and found that miR-338-3p, miR-142-5p and miR-142-3p, miR-216a-5p, miR-223-3p, miR-27a-5p, and miR-378g were significantly correlated with off-pump cardiac index values. It was suggested that these miRNAs might contribute to molecular regulation of reverse remodeling and heart recovery mechanisms in HF patients [89]. Therefore, circulating microRNAs offer a promising novel diagnostic and prognostic biomarker as well as a new therapeutic target for HF patients.

## 7. Oxidative Stress Associated miRNAs with Heart Failure

Oxidative stress plays a significant role in cardiovascular diseases, such as hypertension, cardiac hypertrophy, atherosclerosis, AMI, and HF. Oxidative stress represents a persistent imbalance between the production and the compensation of ROS. Excessive accumulation of ROS can damage proteins, lipids, and DNA of a cell which leads to cellular apoptosis and cell death. Studies have shown that plasma miR-1 level is significantly increased in HF and critically involved in the progression of HF through cardiomyocyte apoptosis and cell death. Administration of anti-miR-1 significantly decreased ROS and oxidative stress susceptibility through SOD1, Gclc, and G6PD and markedly increased heart function in mice model [90]. MiR-181c significantly improved heart function by suppression of ROS production through targeting the 3′-end of mt-COX-1 (cytochrome c oxidase subunit 1) in HF mice [35].

MiR-25 was markedly elevated in response to oxidative stimulation in cardiomyocytes. However, overexpression of miR-25 protected cardiomyocytes against oxidative damage by downregulating mitochondrial calcium uniporter (MCU) and enhanced cardiomyocyte activity [91]. Moreover, miR-21 protects against the oxidative stress induced injury on cardiac myocytes through its target gene programed cell death 4 (PDCD4) [26]. Furthermore, miR-499 protects cardiomyocytes from oxidative stress induced apoptosis via its effects on Pdcd4, Pacs2, and SOX6 [92, 93]. In addition, the expression of miR-200c is significantly elevated by oxidative stress and markedly inhibits endothelial cell growth and increased cell apoptosis and senescence through downregulation of the zinc finger e-box binding homeobox 1 (ZEB1) and endothelium-dependent relaxations (EDRs), while suppression of miR-200c by anti-miR-200c enhanced cell growth and significantly decreased cell apoptosis. Furthermore, holocytochrome c synthetase (HCCS), caspase-3, cyclooxygenase-2 (COX-2), and Slc25a3 protein expressions are essentially controlled by miR-200c and enormously reduced cardiomyocyte death [29, 94–96]. The expression of miR-144 was significantly downregulated in human failing hearts. However, upregulation of the miR-144 to protect against oxidative stress induced cardiomyocyte death via targeting the CUG triplet repeat-binding protein 2- (CUGBP2-) COX-2 signaling pathway [97, 98].

It has been mentioned that upregulated miR-466h inhibits antiapoptotic genes and induces apoptosis. In contrast, the inhibition of the miR-466h increased the expression levels of bcl2l2, dad1, birc6, stat5a, and smo genes and resulted in increased cell viability and significantly decreased caspase-3/7 activity [99]. Moreover, miR-466h-5p is a member of the miR-297-669 cluster located in intron 10 of Sfmbt2 gene on chromosome 2 and has an important proapoptotic role. Further study showed that the time-dependant activation of miR-466h-5p, miR-669c, and the Sfmbt2 gene followed the inhibition of histone deacetylation caused by glucose deprivation-induced oxidative stress [100]. Circulating miR-17-5p level was considerably altered in HF patients and its level was significantly upregulated in myocardium under oxidative stress. Overexpression of miR-17-5p provoked cardiomyocyte injury with decreased cell viability and enhanced apoptotic cell death induced by H_2_O_2_, while inhibition of miR-17-5p by its anti-miR-17-5p markedly reduced the infarct area and apoptosis and significantly increased cell viability through targeting Stat3 [76, 101, 102].

## 8. Limitations and Future Directions

Up to now, most of the studies evaluating that circulating miRNAs act as a biomarker for HF patients are single-center study using relatively small samples, which results in many divergences between different reports. Therefore, it is necessary to perform multicenter large-scale clinical studies to confirm the potential value of circulating miRNAs as noninvasive diagnostic or prognostic biomarkers for HF patients. However, measurement of circulating miRNAs requires qRT-PCR, which is expensive and time-consuming. Therefore, less expensive and newer techniques to detect circulating miRNA levels more rapidly can be expected in the near future.

So far, most of the studies focus on the role of circulating miRNAs as biomarkers for HF. The information regarding the molecular mechanism of circulating miRNAs in HF patients is largely unknown. Consequently, it is also essential to elucidate the mechanisms behind the change of circulating miRNAs before application in clinical practice. MicroRNAs are rapidly becoming an exciting pharmacological target in the treatment of cardiovascular disease including HF. However, there are several limitations overcome to promote miRNAs as a possible therapeutic target. Firstly, miRNAs often have hundreds or even thousands of predicted mRNA targets, but, at physiological expression levels, miRNA most likely targets only a little fraction of them. Secondly, because systemic delivery of antagomirs and miRNA mimics affects miRNA expression, generally, effects observed in the heart could also be secondary to effects in other tissues, for example, as a result of changed blood pressure or alteration in the level of circulating hormones. Finally, the most valuable model to study the function of miRNA is genetic deletion, which should exclusively derepress only those mRNAs that are physiologically repressed by the miRNA. Therefore, it is necessary to discover appropriate route of administration with fewer side effects and miRNA biology must be examined through the use of a combination of genetic animal models and pharmacological manipulation as well as making sure of the safeness of anti-miR or mimic before using it in clinical practice.

## 9. Conclusion

In summary, we suggested that circulating miRNAs may have a potential value for the management of HF patients.

## Figures and Tables

**Table 1:** 

MicroRNAs	Regulation	Sources	Association	Methods	Study population	Number of samples	Potential value	Reference
miR-208b, miR-208a, and miR-499	Up	Serum and plasma	NT-proBNP	A miRNA array/qRT-PCR	Human	*n* = 68	Diagnostic/prognostic	[3, 20]
miR-423-5p	Up	Plasma	NT-proBNP	A miRNA array/qRT-PCR	Human	*n* = 113	Diagnostic	[24, 54]
miR-133	Down	Plasma	NT-proBNP	qRT-PCR	Human	*n* = 83	Diagnostic	[55]
miR-103, miR-142-3p, miR-30b, and miR-342-3p	Down	Plasma	NT-proBNP	A miRNA array/qRT-PCR	Human	*n* = 150	Diagnostic	[56]
miR-126	Down	Plasma	NT-proBNP	qRT-PCR	Human	*n* = 60	Diagnostic	[57]
miR-210 and miR-30a	Up	Serum	NT-proBNP	qRT-PCR	Human	*n* = 40	Diagnostic	[58]
miR-192	Up						Prognostic	[59]
miR-26b-5p, miR-145-5p, miR-92a-3p, miR-30e-5p, and miR-29a-3p	Up	Plasma	NT-proBNP	A miRNA array/qRT-PCR	Human	*n* = 96	Prognostic	[60]
miR-483-3p	Up	Plasma	NT-proBNP	Microarray assay	Human	*n* = 92	Prognostic	[61]
